# Diversity unearthed by the estimated molecular phylogeny and ecologically quantitative characteristics of uncultured *Ehrlichia* bacteria in *Haemaphysalis* ticks, Japan

**DOI:** 10.1038/s41598-020-80690-7

**Published:** 2021-01-12

**Authors:** Hongru Su, Eri Onoda, Hitoshi Tai, Hiromi Fujita, Shigetoshi Sakabe, Kentaro Azuma, Shigehiro Akachi, Saori Oishi, Fuyuki Abe, Shuji Ando, Norio Ohashi

**Affiliations:** 1 Laboratory of Microbiology, Graduate Program in Pharmaceutical and Nutritional Sciences, Department of Food and Nutritional Sciences, School of Food and Nutritional Sciences, Integrated Graduate School of Pharmaceutical and Nutritional Sciences, University of Shizuoka, 52-1 Yada, Suruga-ku, Shizuoka, 422-8526 Japan; 2 Mahara Institute of Medical Acarology, Tokushima, 779-1510 Japan; 3 Department of Medicine and Infectious Disease, Ise Red Cross Hospital, Ise city, Mie, 516-8512 Japan; 4 Mie Prefecture Health and Environment Research Institute, Yokkaichi, Mie 512-1211 Japan; 5 Department of Microbiology, Shizuoka Institute of Environment and Hygiene, Shizuoka, 420-8637 Japan; 6 National Institute of Infectious Diseases, Shinjuku-ku, Tokyo, 1620052 Japan

**Keywords:** Infectious-disease epidemiology, Infectious diseases

## Abstract

*Ehrlichia* species are obligatory intracellular bacteria transmitted by arthropods, and some of these species cause febrile diseases in humans and livestock. Genome sequencing has only been performed with cultured *Ehrlichia* species, and the taxonomic status of such ehrlichiae has been estimated by core genome-based phylogenetic analysis. However, many uncultured ehrlichiae exist in nature throughout the world, including Japan. This study aimed to conduct a molecular-based taxonomic and ecological characterization of uncultured *Ehrlichia* species or genotypes from ticks in Japan. We first surveyed 616 *Haemaphysalis* ticks by *p28*-PCR screening and analyzed five additional housekeeping genes (*16S rRNA*, *groEL*, *gltA*, *ftsZ*, and *rpoB*) from 11 *p28*-PCR-positive ticks. Phylogenetic analyses of the respective genes showed similar trees but with some differences. Furthermore, we found that V1 in the V1–V9 regions of *Ehrlichia 16S rRNA* exhibited the greatest variability. From an ecological viewpoint, the amounts of ehrlichiae in a single tick were found to equal approx. 6.3E+3 to 2.0E+6. Subsequently, core-partial-RGGFR-based phylogenetic analysis based on the concatenated sequences of the five housekeeping loci revealed six *Ehrlichia* genotypes, which included potentially new *Ehrlichia* species. Thus, our approach contributes to the taxonomic profiling and ecological quantitative analysis of uncultured or unidentified *Ehrlichia* species or genotypes worldwide.

## Introduction

*Ehrlichia* species in the family *Anaplasmataceae* are obligatory intracellular bacteria with a lifecycle that is horizontally transmitted between individual wild mammals as natural hosts through arthropod vectors, particularly ticks^1–3^. Some species infect mostly monocytes/macrophages or granulocytes in humans and livestock through tick bites and cause a febrile illness called ehrlichiosis^1–5^. To the best of our knowledge, complete genome sequences or draft genome assembly has been obtained only from *Ehrlichia* species or genotypes isolated and maintained in culture with mammalian or tick cell lines, such as *E. chaffeensis* (human ehrlichiosis), *E. canis* (canine ehrlichiosis), *E. ruminantium* (ruminant heartwater), *E. muris* subsp. *muris*, *E. muris* subsp. *eauclairensis* (human ehrlichiosis), *Ehrlichia* sp. HF (a nomenclature has not yet been conferred), and *E. minasensis*. The genome information of all *Ehrlichia* spp. is summarized in Supplementary Table S1. A core genome-based phylogenetic analysis based on genome sequences revealed the taxonomic status of such cultured *Ehrlichia* species in the family *Anaplasmataceae*^6,7^. However, many studies have investigated the gene detection of uncultured *Ehrlichia* species or genotypes from ticks as well as wild mammals worldwide, including Japan. Representatives of the published reports are summarized in Supplementary Table S2. Because these previous studies used the individual PCR primers for respective target genes (e.g., *16S rRNA*, *groEL*, *gltA*, *rpoB*, and *dsb*)^8–19^, all *Ehrlichia* species or genotypes detected cannot be directly compared or classified. Moreover, although the amount of ehrlichiae in a single tick is thought to be significant information from an ecological viewpoint, such as tick transmission and surveillance, these data remain elusive. Specific quantification data for some identified *Ehrlichia* species obtained by quantitative real-time PCR (qPCR) have previously been reported^18,20–22^, but these assays are not available for other or unknown *Ehrlichia* bacteria in ticks or wild mammals. This study aimed to (1) identify uncultured *Ehrlichia* bacteria from ticks in Japan by specific *p28*-PCR screening, (2) characterize the detailed diversity unearthed by estimated molecular phylogeny based on five additional housekeeping genes, and (3) quantify the copy number of ehrlichiae in a single tick by a newly developed qPCR assay. Therefore, this study is expected to provide an improved understanding of the taxonomic status and ecologically quantitative properties of uncultured *Ehrlichia* bacteria in a tick, and our approach will be applicable and contribute to the comprehensive taxonomic and ecological profiling of unidentified *Ehrlichia* species or genotypes worldwide

## Results

### *Ehrlichia* detection from *Haemaphysalis* ticks by *p28*-PCR screening

**Figure 1:**
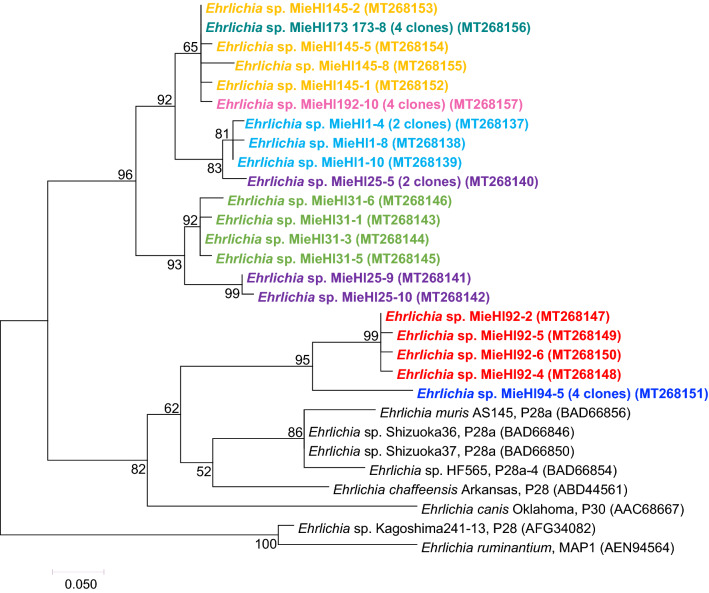
Phylogenetic tree of *p28* clones based on 88–91 amino acid sequences from uncultured *Ehrlichia* bacteria and their close relatives. The tree was constructed using the maximum likelihood method with the Jones–Taylor–Thornton model and “all sites” for gaps/missing data treatment. The numbers on the branch node of the tree indicate bootstrap values from 1000 replications. The scale bar indicates the evolutionary distance. The *p28* clones of the uncultured *Ehrlichia* from a tick individual are indicated by characters with the same color. The accession numbers are in parentheses.

We collected 616 unfed ticks in endemic areas for Japanese spotted fever as well as *Anaplasma phagocytophilum*-infected ticks inhabiting these areas in Japan^23^. These ticks were morphologically identified as *Haemaphysalis hystricis* (n = 48), *H. cornigera* (n = 29), *H. kitaokai* (n = 3), *H. flava* (n = 21), *H. formosensis* (n = 32) and *H. longicornis* (n = 483) (Table 1). For PCR screening, we selected *p28* multigenes of *Ehrlichia* spp. as target genes because *p28* paralogous genes are highly specific for *Ehrlichia* spp.^9,24^. As determined by *p28*-PCR screening, 11 out of 616 ticks (1.8%; nine *H. longicornis* individuals [six males and three females), one *H. hystricis* individual (female) and one *H. flava* individual (nymph)] were found to be positive for ehrlichial infection by PCR (Table 1). Statistical analysis using Fisher’s extract test showed that the ratio of positive adult ticks (males + females) was significantly higher than that of positive nymphs (*p* = 0.003). Three tick species, *H. cornigera*, *H. kitaokai*, and *H. formosensis*, yielded negative PCR results. The amplicons of *p28* multigenes from eight positive ticks were successfully cloned, and the recombinant clones were randomly selected and sequenced. However, three amplicons from a female *H. longicornis* individual, a female *H. hystricis* individual, and a *H. flava* nymph could not be cloned because the amount of gel-purified amplicons was extremely low. A phylogenetic tree was constructed based on the deduced amino acid sequences from 21 *p28-*different clones obtained in this study and eight closely related clones from the other *Ehrlichia* spp. (similarities of 61.4–98.9% among all *p28* clones within the tree, Fig. 1). In the tree, 16 different *p28* clones from six *H. longicornis* ticks were located in a large clade and far from the other *Ehrlichia* bacteria, such as *Ehrlichia* sp. HF565 P28a-4 (70.6–72.9%), *Ehrlichia* sp. Shizuoka36 P28a (69.4–72.9%), *Ehrlichia* sp. Shizuoka37 P28a (69.4–72.9%), *E. muris* AS145 P28a (65.9–69.4%), *E. chaffeensis* Arkansas P28 (64.8–69.7%), *E. canis* Oklahoma P30 (65.6–68.9%), *E. ruminantium* MAP-1 (65.9–69.4%), and *Ehrlichia* sp. Kagoshima241-13 P28 (62.9–65.5%). The five remaining clones from two *H. longicornis* ticks (designated MieHl92 and MieHl94) were positioned into another clade most closely associated with *Ehrlichia* sp. Shizuoka36 P28a (76.7–81.0%), *Ehrlichia* sp. Shizuoka37 P28a (76.7–81.0%), *Ehrlichia* sp. HF565 P28a-4 (76.7–79.8%) and *E. muris* AS145 P28a (75.6–77.4%), followed by *E. chaffeensis* Arkansas P28 (75.6–79.8%, a human pathogen).

**Table 1:** Detection of uncultured *Ehrlichia* bacteria from ticks by *p28*-PCR screening.

Tick species*	No. tested				No. of *Ehrlichia* positive (%)			
	Male	Female	Nymph	Total	Male	Female	Nymph	Total
*H. hystricis*	15	13	20	48	0 (0)	1 (7.7)	0 (0)	1 (2.1)
*H. cornigera*	2	0	27	29	0 (0)	0 (0)	0 (0)	0 (0)
*H. kitaokai*	1	2	0	3	0 (0)	0 (0)	0 (0)	0 (0)
*H. flava*	1	2	18	21	0 (0)	0 (0)	1 (5.6)	1 (4.8)
*H. formosensis*	8	15	9	32	0 (0)	0 (0)	0 (0)	0 (0)
*H. longicornis*	111	109	263	483	6 (5.4)	3 (2.8)	0 (0)	9 (1.9)
Total	138	141	337	616	6 (4.3)**	4 (2.8)**	1 (0.3)	11 (1.8)

*H: *Haemaphysalis.*

**Statistical significance of the 10 positive adult ticks (females + males) *vs* one positive nymph using Fisher’s extract test (*p* = 0.003).

### Multiple gene sequence analysis

**Figure 2:**
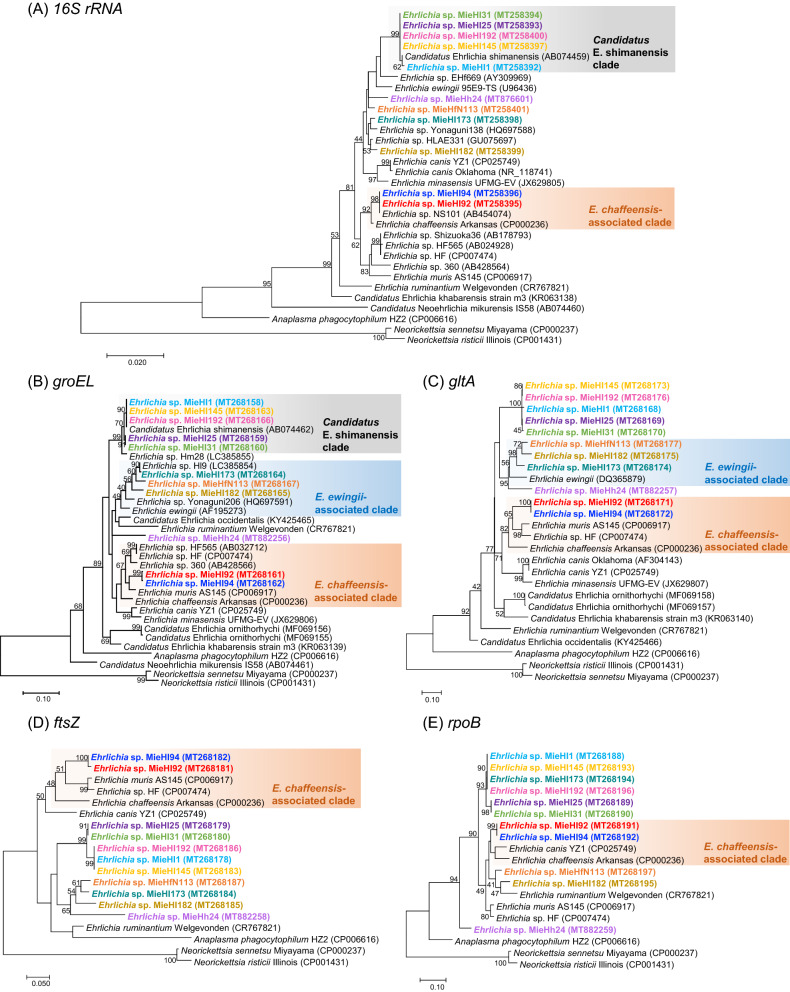
Phylogenetic analyses of five housekeeping genes from uncultured *Ehrlichi*a bacteria based on nucleotide sequences. (**A**) *16S rRNA* (1396–1397 bp), (**B**) *groEL* (427 bp), (**C**) *gltA* (430 bp), (**D**) *ftsZ* (321 bp), and (**E**) *rpoB* (271 bp). These trees were constructed using the maximum likelihood method with the Kimura two-parameter model and “complete deletion” for gaps/missing data treatment. The bootstrap values were obtained from 1000 replications. Bootstrap values higher than 40 are shown in the tree branches. The scale bar indicates the evolutionary distance, and the accession number of each sequence is shown in parentheses. The uncultured *Ehrlichia* bacteria from tick individuals in this study are shown by characters with the same colors on the respective trees of five genes.

To further characterize the uncultured *Ehrlichia* bacteria, we designed PCR primers based on DNA sequences conserved among five housekeeping genes of 16S ribosomal RNA (*16S rRNA*), heat-shock protein (*groEL*), citrate synthase (*gltA*), bacterial cell division protein (*ftsZ*) and RNA polymerase ß-subunit (*rpoB*) from the published genome sequences of *Ehrlichia* spp. The PCR primers are shown in Supplementary Table S3. Through conventional PCR with these primers, we successfully obtained the amplicons and their sequences from all DNA samples of 11 *p28*-positive ticks. The previously reported primers^10^, which were confirmed to be available for our purpose by in silico analysis (Supplementary Table S3), could amplify an additional part of *groEL* DNA. The phylogenetic trees were independently constructed based on the obtained sequences (without primer regions) of *16S rRNA* (1396–1397 bp), *groEL* (2 concatenated sequences, 427 bp), *gltA* (430 bp), *ftsZ* (321 bp), and *rpoB* (271 bp) from the uncultured ehrlichiae in 11 *p28*-positive ticks and from the other *Anaplasmataceae* bacteria (Fig. 2). In all the trees, five of 11 uncultured ehrlichiae from *H. longicornis* ticks (designated MieHl1, MieHl25, MieHl31, MieHl145, and MieHl192) were positioned into a large clade, which showed that they are closely related to *Candidatus* Ehrlichia shimanensis in the trees of *16S rRNA* and *groEL* (termed *Candidatus* E. shimanensis-associated clade, 99.9% and 99.1–100.0% similarities with *Candidatus* E. shimanensis *16S rRNA* and *groEL*, respectively). Two uncultured ehrlichiae (MieHl92 and MieHl94) in all the trees were grouped into *E. chaffeensis*-associated clades, which included *Ehrlichia* sp. HF and *E. muris* in the tree based on *groEL*, *gltA*, and *ftsZ* and *E. canis* in the tree based on *rpoB* (99.4%, 92.7–93.2%, 82.3%, 87.2–87.5% and 91.5% similarities with human pathogenic *E. chaffeensis 16S rRNA*, *groEL*, *gltA*, *ftsZ*, and *rpoB*, respectively). Three uncultured ehrlichiae (MieHl173 and MieHl182 from *H. longicornis* and MieHfN113 from *H. flava*) were classified into an *E. ewingii*-associated clade in the trees based on *groEL* and *gltA* (93.0–93.7% and 84.8–86.7% similarities with *E. ewingii groEL* and *gltA*, respectively), although ehrlichiae MieHl173 in the *rpoB* tree was grouped into a different large clade consisting of six uncultured ehrlichiae. Additionally, *Ehrlichia* sp. MieHh24 detected from *H. hystricis* was independently located and far from the 10 other uncultured ehrlichiae in all five trees. Thus, a phylogenetic analysis using a single target gene appears to provide insufficient resolution for the taxonomic classification of unknown *Ehrlichia* species or genotypes.

### Polymorphic site of variable regions on *Ehrlichia 16S rRNA*

**Figure 3:**
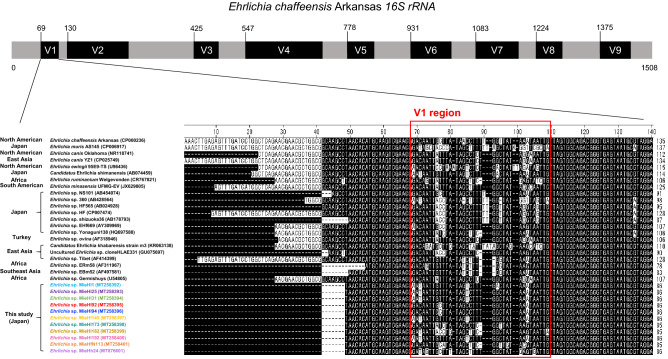
Alignment of the V1 region of *16S rRNA* sequences from 33 *Ehrlichia* bacteria, including 11 uncultured ehrlichiae, in this study. The V1 region of the *Ehrlichia 16S rRNA* alignment is boxed with a red line.

The *16S rRNA* sequences that are generally used for the taxonomic classification of bacteria are known to have nine hypervariable regions (V1–V9)^25^. The almost full-length *16S rRNA* sequences (approx. 1.4 kb) from 33 ehrlichiae, including 11 uncultured ehrlichiae in this study and 22 other *Ehrlichia* spp., were aligned to characterize the sequence variation (Supplementary Fig. S1). In the V1–V9 regions, the V1 region was found to be exhibit the most diversity among 33 *Ehrlichia 16S rRNA* sequences (Fig. 3). The calculated numbers of polymorphic sites in the V1–V9 regions confirmed showed that the V1 region had the highest number of these sites (Table 2).

**Table 2:** Polymorphic sites of V1–V9 regions in *Ehrlichia 16S rRNA*. *The position on the alignment with gaps in Fig. 3 and Supplementary Fig. S1 is shown here.

Variable region	*E. chaffeensis* Arkansas *16S rRNA*		Position*	Polymorphic sites (%)
	Start	End		
V1	69	104	69–109	34 (82.9)
V2	130	270	135–276	16 (11.3)
V3	425	481	431–487	5 (8.8)
V4	547	725	553–731	14 (7.8)
V5	778	846	785–853	5 (7.2)
V6	931	1025	939–1033	13 (13.7)
V7	1083	1183	1091–1191	5 (5.0)
V8	1224	1283	1232–1291	13 (21.7)
V9	1375	1445	1383–1453	6 (8.5)

### Estimation of the copy numbers of uncultured *Ehrlichia* bacteria in a single tick

We first performed real-time PCR targeting *groEL*, *gltA*, *ftsZ* and *rpoB* using serially diluted genomic DNA extracted from *E. chaffeensis*-infected DH82 cells. As a result, we selected *gltA* as the target that appears to be the most adequate in terms of specificity and sensitivity for our purpose. Calibration curves were then prepared by qPCR using dilutions of the standard *gltA* amplicons as the DNA templates in each experiment (a representative curve is shown in Supplementary Fig. S2). Based on this analysis, the limit of detection of the qPCR assay was estimated to equal three copies. Subsequently, the *gltA* copy numbers corresponding to *Ehrlichia* numbers in 1 µL of *Ehrlichia*-positive tick samples were determined to equal 2.1E+2 to 6.6E+4 (Table 3). Based on the DNA elution volumes, the number of ehrlichiae in a single tick was ultimately estimated to be in the approx. range of 6.3E+3 to 2.0E+6.

**Table 3:** The *gltA* copy numbers of uncultured *Ehrlichia* bacteria in ticks by qPCR.

Tick ID	Tick species*	Stage	Mean *gltA* copies/µL	Estimation of *gltA* copies/tick**
Hl1	*H. longicornis*	Male	2.0E+3	6.1E+4
Hl25		Male	1.2E+4	3.5E+5
Hl31		Male	4.9E+3	1.5E+5
Hl92		Male	6.0E+3	1.8E+5
Hl94		Male	8.1E+3	2.4E+5
Hl145		Female	9.1E+3	2.7E+5
Hl173		Male	3.4E+4	1.0E+6
Hl182		Female	6.6E+4	2.0E+6
Hl192		Female	5.5E+4	1.7E+6
HfN113	*H. flava*	Nymph	3.3E+4	9.9E+5
Hh24	*H. hystricis*	Female	2.1E+2	6.3E+3

*H: *Haemaphysalis*.

**The *gltA* copy numbers in a single tick were estimated by the multiplication of mean *gltA* copies/µL determined and DNA elution volume (30 µL).

### Taxonomic characteristics of uncultured *Ehrlichia* bacteria unearthed through estimated molecular phylogeny

**Figure 4:**
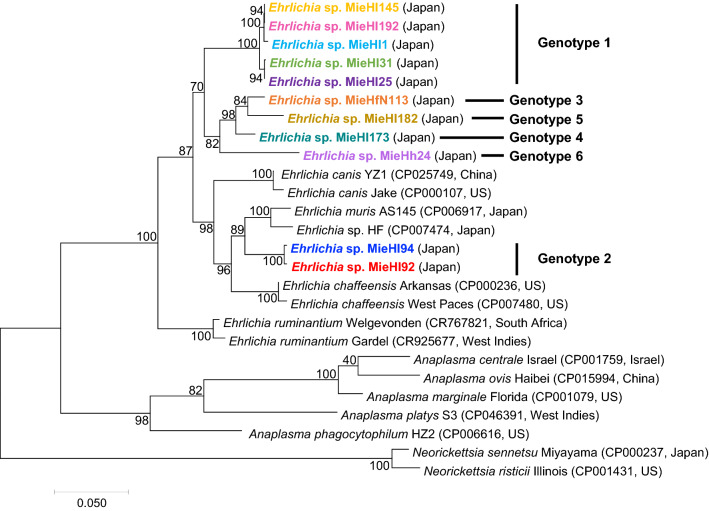
Core-partial-RGGFR-based phylogenetic analysis of uncultured *Ehrlichia* bacteria and cultured *Ehrlichia* species with genome sequence data. Tree based on five housekeeping genes (*16S rRNA*-*groEL*-*gltA*-*ftsZ*-*rpoB*) of uncultured *Ehrlichia* bacteria in this study and cultured *Ehrlichia* species for which complete genome sequence data have previously been obtained were constructed using the maximum likelihood method with the Kimura two-parameter model and “complete deletion” for gap/missing data treatment. The bootstrap values from 1000 replications are shown on the branch nodes. The scale bar indicates the evolutionary distance. The uncultured *Ehrlichia* bacteria in this study are shown by the colored characters, and their “genotypes” that were estimated based on the core-partial-RGGFR alignment and summarized in Table 4 are shown on the right side. The accession numbers and the location of isolated or identified *Ehrlichia* species or genotypes are shown in parentheses.

The taxonomic status of the uncultured ehrlichiae without genome sequence data was unearthed via estimated molecular phylogeny through a core-partial-RGGFR-based phylogenetic analysis based on the concatenated sequences of five housekeeping loci in the order *16S rRNA*-*groEL*-*gltA*-*ftsZ*-*rpoB* (total length of 2845–2846 bp) using the closely related *Anaplasmataceae* bacteria with genome sequence data available in the database (Fig. 4). The tree revealed that the uncultured ehrlichiae were located separately into two clades: a large independent clade (nine ehrlichiae) and a clade (two ehrlichiae) associated mainly with *E. muris* followed by *Ehrlichia* sp. HF. The similarities among the 11 uncultured *Ehrlichia* bacteria were 90.7–100% (*Ehrlichia* sp. MieHl145 and MieHl192 showed 100% similarity). Additionally, the 11ehrlichiae showed similarities of 90.4–94.7% with *E. muris* str. AS145, 90.4–94.6% with *Ehrlichia* sp. HF, 90.4–94.0% with *E. chaffeensis* str. Arkansas, 90.1–93.6% with *E. chaffeensis* str. West Paces, 90.3–93.1% with *E. canis* str. YZ1, 89.8–92.5% with *E. canis* str. Jake, 89.2–91.2% with *E. ruminantium* str. Gardel and 89.5–91.5% with *E. ruminantium* str. Welgevonden. Based on these taxonomic analyses, six *Ehrlichia* genotypes, including potentially new *Ehrlichia* species, were found to exist in Japan and are summarized in Table 4.

**Table 4:** Genotypes of uncultured *Ehrlichia* bacteria in the current study based on the similarities of the core-partial-RGGFR alignment. Under consideration based on the bacterial species criteria (96.8%) due to core genome alignments as previously described^7^, the 11 uncultured *Ehrlichia* members could be divided into six genotypes, probably including novel *Ehrlichia* species.

Species or genotype:bacterial ID	Closest relatives (bacterial ID)	Similarity %	Source*	References
*E. chaffeensis* Arkansas	*E. chaffeensis* West Paces	99.4	Human	^42^
*E. canis* YZ1	*E. canis* Jake	99.3	Dog	^45,46^
*E. ruminantium* Welgevonden	*E. ruminantium* Gardel	98.8	*Amblyomma hebraeum*, Goat	^47,48^
*E. muris* AS145	*Ehrlichia* sp. HF	97.3	Wild rodent, *Ixodes ovatus*	^36^
Genotype 1: *Ehrlichia* sp. MieHl1, MieHl25, MieHl31, MieHl145, MieHl192	*E. muris* AS145, *Ehrlichia* sp. HF, *Candidatus* E. shimanensis** (*Ehrlichia* sp. MieHl1, MieHl25, MieHl31, MieHl145, MieHl192)	92.0–92.1, 92.1–92.4 (99.1–100)	*H. longicornis*	This study
Genotype 2: *Ehrlichia* sp. MieHl92, MieHl94	*E. muris* AS145, *Ehrlichia* sp. HF (*Ehrlichia* sp. MieHfN113)	94.7, 94.5–94.6 (92.4–92.5)	*H. longicornis*	This study
Genotype 3: *Ehrlichia* sp. MieHfN113	*E. muris* AS145, *Ehrlichia* sp. HF, *E. chaffenesis*, *E. canis* (*Ehrlichia* sp. MieHl173)	92.0–92.6 (97.0)	*H. flava*	This study
Genotype 4: *Ehrlichia* sp. MieHl173	*E. muris* AS145, *Ehrlichia* sp. HF, *E. chaffenesis*, *E. canis* (*Ehrlichia* sp. MieHfN113)	91.5–92.5 (97.0)	*H. longicornis*	This study
Genotype 5: *Ehrlichia* sp. MieHl182	*E. muris* AS145*, Ehrlichia* sp. HF, *E. chaffenesis, E. canis* (*Ehrlichia* sp. MieHfN113)	90.5–92.7 (96.5)	*H. longicornis*	This study
Genotype 6: *Ehrlichia* sp. MieHh24	*E. muris* AS145, *Ehrlichia* sp. HF, *E. chaffenesis*, *E. canis* (*Ehrlichia* sp. MieHfN113)	89.8–90.4 (92.8)	*H. hystricis*	This study

*H: *Haemaphysalis*.

**Five *Ehrlichia* members in genotype 1 may belong to “*Candidatus* E. shimanensis” that has previously been proposed based on *16S rRNA* and *groEL*^28^.

## Discussion

The present study demonstrated the molecular-based diversity among 11 newly identified *Ehrlichia* members from *Haemaphysalis* ticks (*H. longicornis*, *H. hystricis*, and *H. flava*) through our developed taxonomic profiling approach. The genotypes of these 11 members based on the current phylogenetic analyses are summarized in Table 4 and discussed here. Based on the *16S rRNA* and *groEL* analyses, five *Ehrlichia* sp., MieHl1, MieHl25, MieHl31, MieHl145, and MieHl192 (termed genotype 1), are likely members of the *Candidatus* E. shimanensis group. On the core-partial-RGGFR-based tree, two *Ehrlichia* sp., MieHl92 and MieHl94 (genotype 2), were mainly associated with *E. muris*, *Ehrlichia* sp. HF followed by *E. chaffeensis*. The analyses of *groEL* and *gltA* revealed that *Ehrlichia* sp., MieHfN113 and MieHl182 (genotypes 3 and 5), might be related to *E. ewingii*. It should be considered that *E. ewingii* infects granulocytes^1,2^, but most *Ehrlichia* members infect monocytes/macrophages in mammalian cells. Biological information regarding such *E. ewingii*-related members will be investigated in future studies. *Ehrlichia* sp. MieHl173 (genotype 4) was located in a large clade in the *rpoB*-based tree but was classified into different clades in the other gene-based trees. *Ehrlichia* sp. MieHh24 (genotype 6) was found to likely be independently located in all trees. A previous study suggested that genomes from bacteria with the same species epithet consistently exhibit more than 96.8% identity in their core genome alignments^7^. Based on this bacterial species criteria (96.8%) due to core genome alignments, we could classify the 11 uncultured *Ehrlichia* members into six genotypes: genotype 1 includes five ehrlichiae (*Ehrlichia* sp. MieHl1, MieHl25, MieHl31, MieHl145, and MieHl192), genotype 2 comprises two ehrlichiae (*Ehrlichia* sp. MieHl92 and MieHl94), genotype 3 includes *Ehrlichia* sp. MieHfN113, genotype 4 consists of *Ehrlichia* sp. MieHl173, genotype 5 includes *Ehrlichia* sp. MieHl182, and genotype 6 consists of *Ehrlichia* sp. MieHh24. These genotypes likely include novel *Ehrlichia* species (Table 4).

In general, most *Ehrlichia* species or genotypes are horizontally transmitted between mammals as natural reservoirs and ticks as arthropod vectors through tick bites. Hence, many tick species have become vector candidates for several *Ehrlichia* species or genotypes. Indeed, previous studies conducted in East Asia have identified various uncultured *Ehrlichia* species or genotypes detected from *Haemaphysalis* ticks, such as *H. longicornis*^8,9,26–28^, *H. flava*^8,27^, and *H. megaspinosa*^27^ in Japan, *H. longicornis*^29,30^, *H. flava*^29^, and *H. hystricis*^31^ in China, and *H. longicornis*^32,33^ in South Korea. Furthermore, different *Ehrlichia* species or genotypes have been detected from *Ixodes persulcatus*^11,12^, *I. ovatus*^11,24,27,34^, *I. granulatus*^35^, and *I. turdus*^27^ in Japan. Among these, *E. muris* subsp. *muris* and *Ehrlichia* sp. HFs, which have been isolated and maintained in culture (the genome information is shown in Supplemental Table S1) are potentially transmitted by *I. persulcatus* and *I. ovatus*, respectively^11,12,24,27,28,34,36^. Thus, the current and previous studies suggest that a single tick species carries several *Ehrlichia* species or genotypes through horizontal transmission. In terms of transmission ability, the variety of *Ehrlichia* species or genotypes is unlikely limitless and dependent on the tick species (probably “regulated due to adaptation in tick species”). Therefore, the global classification of unidentified *Ehrlichia* bacteria in ticks and mammalian hosts will be highly significant and demanded, e.g., as in the current study aiming to perform comprehensive taxonomic profiling without genome sequencing.

The field survey of ehrlichiae in the current study was conducted in a narrow area of the Mie prefecture in Japan, which is a high-risk area for Japanese spotted fever and anaplasmosis^23,37^, because we have confirmed the presence of antibody against *E. chaffeensis* in the sera of some tick-borne-suspected patients with fever of unknown origin in this area. Accordingly, the members of the uncultured *Ehrlichia* genotypes in the current study have become candidate human pathogens. In Taiwan, two cases of human infections with *E. chaffeensis* have been reported^38,39^. The short *16S rRNA* sequence (182 bp) detected from the first patient^38^ was identical to that from all 11 members of the uncultured *Ehrlichia* genotypes as well as *E. chaffeensis*, and the *Ehrlichia 16S rRNA* sequence (345 bp) from the second patient^39^ was only identical to that of *Ehrlichia* sp. MieHl92 and MieHl94 (genotype 2) as well as *E. chaffeensis*. Taken together, the results indicate that the members of genotype 2 that are mainly related to *E. muris* and *Ehrlichia* sp. HF followed by *E. chaffeensis* are the most likely candidates to act as human ehrlichiosis agents in Japan.

We performed *p28*-PCR screening and phylogenetic analysis based on the amino acid sequences of *p28* clones. The *p28* multigenes encode a major outer membrane protein (OMP) family consisting of 22 protein species^5,40–42^. In general, the spontaneous mutation of bacterial OMP genes, including *p28* multigenes, occurs frequently due to the pressure of environmental bias, e.g., the repeated host changes needed for *Ehrlichia* survival due to horizontal transmission between mammals and ticks. From this perspective, we constructed a phylogenetic tree of *p28* (OMP) clones using the sequences of amino acids rather than nucleotides to confirm the phenotypic variation. Additionally, *p28* clone data were not used for the multiple loci-based construction of trees for taxonomic profiling of the uncultured *Ehrlichia* bacteria in Fig. 4 because OMP gene mutations are thought to be undergo more rapid accumulation than those of housekeeping genes due to environmental bias.

*16S rRNA* sequences are available and frequently used for bacterial taxonomy, and the bacterial *16S rRNA* sequences contain V1–V9 regions^25^. In *16S rRNA*-based metagenomics, the V3–V4 regions have frequently been used as a target in bacterial identification at the family level and even the genus level^43^. However, the V1 region of *Ehrlichia* members is found to exhibit the highest variability, which suggests that the V1 region benefits the identification of *Ehrlichia* species or genotypes.

From an ecological perspective, the tick stage associated with *Ehrlichia* transmission is likely the “adult stage” based on the statistical significance between the positive ratios of adults and nymphs. It is possible that nymphs acquire *Ehrlichia* organisms from natural host mammals through horizontal transmission and become infected adult ticks through molting. In contrast, the nymph stage plays an important role in *A. phagocytophilum* transmission in Japan as described previously^23^. The number of ehrlichiae in a single infected tick that was estimated by the newly developed qPCR method was found to be in the wide range of 6.3E+3 to 2.0E+6. Some infected ticks can unexpectedly carry a large number of ehrlichiae. To the best of our knowledge, this study provides the first estimate of the number of ehrlichiae in an individual tick in nature by qPCR.

As mentioned above, the current study provides significant information regarding the taxonomic and ecological characteristics of uncultured *Ehrlichia* bacteria from *Haemaphysalis* ticks in Japan. Our approach is applicable and contributes to the detailed molecular-based characterization and the surveillance of unidentified or uncultured *Ehrlichia* species or genotypes worldwide in future studies.

## Materials and methods

### Tick collection and DNA preparation

A total of 616 unfed ticks were collected by flagging at 13 sites (within a square area located from 34°29′33.6″ to 34°36′59.9″N and 136°53′88.8″ to 136°62′48.6″E) of the forests or weedy regions in Shima Peninsula at the Mie prefecture, Japan, during June and July of 2018. These areas are known to be endemic for Japanese spotted fever^37^ and at high risk for anaplasmosis^23^. The ticks obtained were morphologically identified as shown in Table 1 and maintained in sterile tubes under continuous humidity at 16 °C. For DNA preparation, these live ticks were washed with 0.12% sodium hypochlorite solution followed by 70% ethanol supplemented with 1% povidone-iodine solution for 10 min during each disinfection cycle to avoid contamination by soil bacteria on the body surface of the ticks and rinsed with phosphate-buffered saline (PBS, pH 7.4). The ticks were then individually dissected using a sterile and disposable blade, and total DNA was extracted from the whole tissues of each tick using the InstaGene Matrix Kit (Bio-Rad Laboratories, Hercules, CA, USA) or QIAamp DNA Mini Kit (QIAGEN, Hilden, Germany) according to the manufacturer’s instructions. The final elution volume for DNA extraction was 30 µL, and the DNA samples were stored at − 30 °C until use.

### *Ehrlichia* detection by *p28*-PCR screening from ticks

The DNA samples from all the ticks were individually screened by conventional PCR targeting *p28*-paralougaous genes, which are known as a *p28*-multigene family specific for *Ehrlichia* members^9,24^. The primers are shown in Supplementary Table S3, and nested PCR was conducted in a 25-µL reaction mixture containing 12.5 µL of 2× GoTaq (Promega, USA), 400 nM of each primer, and 1 or 2 µL of the DNA template under previously-described conditions^24^. The *p28* amplicons obtained were subjected to gel-purification using a Wizard SV Gel and PCR Clean-Up System Kit (Promega, USA) and cloned into the pCR2.1 vector using the TA Cloning Kit (Thermo Fisher Scientific, Waltham, MA, USA). The recombinant plasmids were then introduced into *Escherichia coli* DH5α cells (Toyobo Co., Ltd., Osaka, Japan). The randomly selected recombinant *p28* clones were sequenced and phylogenetically analyzed as described below. The recombinant DNA experiment performed in this study was approved by the Committee of University of Shizuoka, Japan (No. 661-2303).

### Housekeeping gene sequencing

The uncultured ehrlichiae from the *p28*-positive PCR samples were further characterized by conventional PCR targeting five housekeeping genes, namely, the *16S rRNA*, *groEL*, *gltA*, *ftsZ* and *rpoB* genes. The primers and amplicon sizes are shown in Supplementary Table S3. Nested PCR was performed in a 25-µL reaction mixture containing GoTaq (Promega, USA), each primer, and 1 or 2 µL of the DNA template as described above. The PCR program was 94 °C for 3 min followed by 45 cycles of 94 °C for 30 s, 55 °C for 1 min, and 72 °C for 1 min, and 72 °C for 10 min. The amplicons were then purified using a Wizard SV Gel and PCR Clean-Up System Kit (Promega, USA), sequenced directly and phylogenetically analyzed as described below.

### Quantitative real-time PCR (qPCR)

To estimate the number of uncultured *Ehrlichia* bacteria in each tick, we first investigated the specificity and sensitivity by real-time PCR targeting *groEL*, *gltA*, *ftsZ* and *rpoB* using the KOD SYBR qPCR Mix (Toyobo Co., Ltd., Osaka, Japan). Genomic DNA was extracted from a culture of DH82 cells infected with the *E. chaffeensis* Arkansas strain (kindly provided by Dr. Robert F Massung at the Centers for Disease Control and Prevention, USA, in 2003) using a QIAamp DNA Mini Kit (QIAGEN, Hilden, Germany) and serially diluted. For real-time PCRs, internal primers of the respective genes for nested PCR shown in Supplementary Table S3 were used. The reaction mixtures were prepared in a volume of 25 µL, which included 1 µL of the sample DNA or serially diluted standard DNA, 400 nM of each primer, and 12.5 µL of KOD SYBR qPCR Mix (Toyobo Co., Ltd., Osaka, Japan). The real-time PCR program consisted of 98 °C for 2 min followed by 45 cycles of 98 °C for 10 s, 60 °C for 30 s, and 68 °C for 40 s. qPCR was performed using a Thermal Cycler Dice Real Time System II (Takara Bio, Shiga, Japan). Based on the results, we selected *gltA* as the target for the quantification of uncultured ehrlichiae in ticks because this gene appears to be the most adequate target in terms of specificity and sensitivity. For quantification, the DNA template prepared using genomic DNA extracted from *E. chaffeensis*-infected DH82 cells was subjected to PCR using external primers of *gltA* (Eh_gltA_112_F1 and Eh_gltA_686_R1 in Supplementary Table S3). The amplicon was subjected to gel purification, and the *gltA* copy number was calculated based on the concentrations of the amplicon using Qubit dsDNA HS Assay Kits (Thermo Fisher Scientific, Waltham, MA, USA). The purified amplicon was serially diluted (3 × 10^7^, 3 × 10^6^, 3 × 10^5^, 3 × 10^4^, 3 × 10^3^, 3 × 10^2^, 3 × 10^1^, 3 × 10^0^, and 3 × 10^–1^ copies/µL) and used as standard DNA. qPCR with the serially diluted standards and the DNA samples was performed using internal primers of *gltA* (Eh_gltA_137_F2 and Eh_gltA_614_R2 in Supplementary Table S3). The *gltA* copy number corresponding to the *Ehrlichia* number per 1 µL of each sample was determined based on the calibration curve of the standard DNAs (Supplementary Fig. S2). The copy number of *Ehrlichia* in a single tick was estimated from a DNA elution volume of 30 µL.

### In silico analysis and statistical analysis

The similarity of the nucleotide and amino acid sequences among uncultured *Ehrlichia* members investigated in this study and the related bacteria existing in GenBank was calculated using MegAlign of DNASTAR software in Lasergene version 14 (DNASTAR, Madison, WI, USA). For phylogenetic analysis, the uncultured *Ehrlichia* sequences were aligned with reference sequences using ClustalW (default parameters) within the MEGA program (version 7.0.26). Based on the sequence alignment, phylogenetic trees were constructed using the maximum likelihood method with the Jones–Taylor–Thornton model (using the default parameters except “all sites” for gaps/missing data treatment) for the amino acid sequences of *p28* clones and using the Kimura two-parameter model (using the default parameters except “complete deletion” for gaps/missing data treatment) for the nucleotide sequences of respective genes. The core-partial-RGGFR-based phylogenetic tree was constructed using the concatenated sequences in the order *16S rRNA*-*groEL*-*gltA*-*ftsZ*-*rpoB* (length of 2845–2846 bp) using the same procedure. Bootstrap values were obtained with 1000 replicates, and values higher than 70 were considered to indicate good confidence^44^. A statistical analysis between positive adult ticks and positive nymphs was performed using Fisher’s extract test in R (R Core Team 2020, Version 4.0.2, https://www.R-project.org/).

### Accession numbers in the GenBank

The sequences of *p28* clones and the respective genes without primer regions obtained in this study were deposited in the GenBank. The accession numbers are MT268137-MT268157 for *p28* clones, MT258392-MT258401 and MT876601 for *16S rRNA*, MT268158-MT268167 and MT882256 for *groEL*, MT268168-MT268177 and MT882257 for *gltA*, MT268178-MT268187 and MT882258 for *ftsZ*, and MT268188-MT268197 and MT882259 for *rpoB*.

## Supplementary Information

Supplementary Information.

## Data Availability

All data supporting the conclusions of this article are included in the paper.
