# Supplementary material for: Diversity unearthed by the estimated molecular phylogeny and ecologically quantitative characteristics of uncultured Ehrlichia bacteria in Haemaphysalis ticks, Japan
Source: Sci Rep. 2021 Jan 12;11:687. doi: 10.1038/s41598-020-80690-7 (PMC7804854; doi:10.1038/s41598-020-80690-7)

SUPPLEMENTARY INFORMATION

Diversity unearthed by the estimated molecular phylogeny and ecologically quantitative characteristics of uncultured *Ehrlichia* bacteria in *Haemaphysalis* ticks, Japan

Hongru Su[1], Eri Onoda[1], Hitoshi Tai[1], Hiromi Fujita[1,2], Shigetoshi Sakabe[3], Kentaro Azuma[3], Shigehiro Akachi[4], Saori Oishi[5], Fuyuki Abe[5], Shuji Ando[6] and Norio Ohashi[1*]

[1]Laboratory of Microbiology, Graduate Program in Pharmaceutical and Nutritional Sciences, Integrated Graduate School of Pharmaceutical and Nutritional Sciences, University of Shizuoka, Shizuoka 422-8526, Japan.

[2]Mahara Institute of Medical Acarology, Tokushima 779-1510, Japan.

[3]Department of Medicine and Infectious Disease, Ise Red Cross Hospital, Ise city, Mie 516-8512, Japan.

[4]Mie Prefecture Health and Environment Research Institute, Mie 512-1211, Japan.

[5]Department of Microbiology, Shizuoka Institute of Environment and Hygiene, Shizuoka 420-8637, Japan.

[6]National Institute of Infectious Diseases, Shinjuku-ku, Tokyo, 1620052, Japan.

*Corresponding author: Norio Ohashi, ohashi@u-shizuoka-ken.ac.jp

Supplementary Table S1: Genome information of *Ehrlichia* strains isolated.

Strain*	Genome type	Size (Mb)	Source	Place of origin	Accession no. (GenBank)	Accession no. (Refseq)	Reference
E. chaffeensis str. Arkansas	Complete	1.18	Human	US	CP000236	NC_007799	42
E. chaffeensis str. West Paces	Complete	1.17	Human	US	CP007480	NZ_CP007480	unpublished
E. canis str. Jake	Complete	1.32	Dog	US	CP000107	NC_007354	45
E. canis str. YZ1	Complete	1.31	Dog	China	CP025749	NC_CP025749	46
E. ruminantium str. Welgevonden	Complete	1.52	*Amblyomma hebraeum*	South Africa	CR767821	NC_005295	47
E. ruminantium str. Gardel	Complete	1.5	Goat	West Indies	CR925677	NC_006831	48
E. muris str. AS145	Complete	1.2	Wild rodent	Japan	CP006917	NC023063	36
E. muris subsp. *eauclairensis* str. EmCRT	Draft	NA	*Ixodes scapularis*	US	LANU01000001-LANU01000003	NA	49
Ehrlichia sp. HF	Complete	1.15	*Ixodes ovatus*	Japan	CP007474	NZ_CP007474	unpublished
E. minasensis str. B11	Draft	NA	Cattle	Brazil	QOHL01000001-QOHL01000055	NA	6,50

*, *E*: *Ehrlichia***.** **, NA: Not available.

Supplementary Table S2: Previous classification of uncultured *Ehrlichia* species or genotypes.

No.	Location	Year	Target gene	Source	Species or genotypes (uncultured)	Reference
1	Japan	2000	*16S rRNA*, *groEL*	*Ixodes ovatus*	*Ehrlichia* sp. Anan	34
2	Japan	2004	*16S rRNA*, *p28*	*Ixodes ovatus*	*Ehrlichia* sp. Shizuoka	24
3	Japan	2006	*16S rRNA*, *groEL*	*Haemaphysalis longicornis*, wild deer	*Candidatus* Ehrlichia shimanensis (*Ehrlichia* sp. TS37), *Ehrlichia* sp. SS15E-L	28
4	Japan	2009	*16S rRNA*, *groEL*	*Ixodes granulatus*	*Ehrlichia* sp. 360	35
5	Japan	2009	*16S rRNA*, *groEL*	Sika deer	*Ehrlichia* sp. NS101	51
6	Japan	2011	*16S rRNA*, *groEL*	*Haemaphysalis longicornis*	*Ehrlichia* sp. Yonaguni 138, *Ehrlichia* sp. Yonaguni 206	26
7	Japan	2019	*16S rRNA*, *groEL*	*Ixodes* sp., *Haemaphysalis* sp.	*Ehrlichia* sp. It20, *Ehrlichia* sp. Io30, *Ehrlichia* sp. Hm28, *Ehrlichia* sp. Hf34, *Ehrlichia* sp. Hl9 etc.	27
8	France	2012	*16S rRNA*, *gltA*	*Argas vespertilionis* (argasid tick)	*Ehrlichia* sp. Av.Bat	52
9	Russia	2015	*16S rRNA*, *groEL*, *gltA*	Wild rodent	*Candidatus* Ehrlichia Khabarensis	53
10	China	2016	*16S rRNA*, *groEL*, *gltA*	*Haemaphysalis longicornis*	Uncultured *Ehrlichia* sp. Daishan	54
11	Australia	2018	*16S rRNA*, *groEL*, *gltA*	*Ixodes ornithorhynchi* (collected from platypuses)	*Candidatus* Ehrlichia ornithorhynchi	55
12	Australia	2017	*16S rRNA*, *groEL*, *gltA*, *map1*	*Amblyomma triguttatum* subsp.	*Candidatus* Ehrlichia occidentalis	56
13	Chile	2019	*16S rRNA*, *groEL*, *dsb*	Magellanic penguins, *Ixodes uriae*,	*Ehrlichia* sp. Magellanica	57
14	Brazil	2012	*16S rRNA*, *groEL*, *gltA*, *dsb*, *gp36*	*Rhipicephalus microplus*	*Ehrlichia minasensis* str. UFMG-EV*	58
15	Philippines	2015	*rpoB*	Dog, *Rhipicephalus sanguineus*	*Ehrlichia canis*-like	19

*, The *Ehrlichia* isolate was subsequently obtained[59] and the draft genome sequence has been deposited in the GenBank (QOHL01000001-QOHL01000055)[50].

Supplementary Table S3: PCR primers used in this study.

Target gene	Primer	Sequence (5'-3')*	Amplicon size (bp)	Reference
p28/omp-1	conP28-F1	ATYAGTGSAAARTAYRTRCCAA	713-714	24
	conP28-R1	TTARAARGYAAAYCTKCCTCC		
	conP28-F2	CAATGGRWGGYCCMAGARTAG	293-294	
	conP28-R2	TTCCYTGRTARGMAAKTTTAGG		
16S rRNA	Eh_16S_21_F1	GGCTCAGAACGAACGCTGG	1474	This study
	Eh_16S_1494_R1	AGCCGCAGGTTCACCTACA		
	Eh_16S_31_F2	GAACGCTGGCGGCAAGCC	1437	
	Eh_16S_1467_R2	GTTACGACTTCACCMTAGTCA		
16S rRNA (for sequencing)	Eh_16S_501_Fseq1	CCAGCAGCCGCGGTAAT		This study, 60
	Eh_16S_501_Rseq1	ATTACCGCGGCTGCTGG		
	Eh_16S_889_Fseq2	AAACTCAAAGGAATTGACGG		
	Eh_16S_889_Rseq2	CCGTCAATTCCTTTGAGTTT		
gltA	Eh_gltA_112_F1	GGRRTRTTAACTTATGATCCAGG	575	This study
	Eh_gltA_686_R1	GCATTYTGYTCATGATCAGCATG		
	Eh_gltA_137_F2	TTATGTCTACTGCTGCTTGTGA	478	
	Eh_gltA_614_R2	TARGAAGAAAYRTCAAACATCATATG		
rpoB	Eh_rpoB_241_F1	AGTTATAGTATTGGTGARCCRCA	581	This study
	Eh_rpoB_821_R1	ARYCTAACWCCYCTRAAYCTATC		
	Eh_rpoB_305_F2	CTGTWCCTATACGTATAGTKYTGCG	319	
	Eh_rpoB_623_R2	TCTARCCAKGAWCCYCTRTARGG		
ftsZ	Eh_ftsZ_242_F1	GTARAGGWGCWGCWGAAGARTCAA	462	This study
	Eh_ftsZ_703_R1	CWGCTTCTCCTGTRCCCATCAT		
	Eh_ftsZ_313_F2	ACTGCYGGAATGGGTGGWGGA	367	
	Eh_ftsZ_679_R2	TTTRCCCATYTCRCTCATTATTGC		
groEL (1)**	Eh_groEL_64_F1	TTRGAAGAYGCWGTAGGATGYAC	530	This study
	Eh_groEL_593_R1	CCWCKRTCAAAYTGCATRCCATC		
	Eh_groEL_107_F2	CYGTAGCWATTRGYAARYCYTATGG	235	
	Eh_groEL_341_R2	CWNAYAATATCTGCHCCAGCAGC		
groEL (2)**	Ehrli-gro67F	GAAGATGCWGTWGGWTGTACKGC	710	10
	Ehrli-gro776R	AGMGCTTCWCCTTCWACRTCYTC		
	Ehrli-gro217F	ATTACTCAGAGTGCTTCTCARTG	365	
	Ehrli-gro581R	TGCATACCRTCAGTYTTTTCAAC		

* Degenerate nucleotides: R = A or G; M = A or C; W = A or T; S = C or G; Y = C or T; K = G or T; H = A, T or C; N = A, C, G or T. ** The concatenated sequences (427 bp) of two *groEL*s without primer regions including 125-bp overlapping sequence were used for phylogenetic analysis as shown in Figure 2 and Figure 4.

Supplementary Figure S1: Alignment of almost full-length *16S rRNA* sequences from 33 *Ehrlichia* bacteria including 11 uncultured *Ehrlichia* members in this study. Blue- or green-highlighted alignment areas with yellow characters (V1-V9) indicate nine variable regions within *Ehrlichia 16S rRNA* estimated based on the previous report[25].

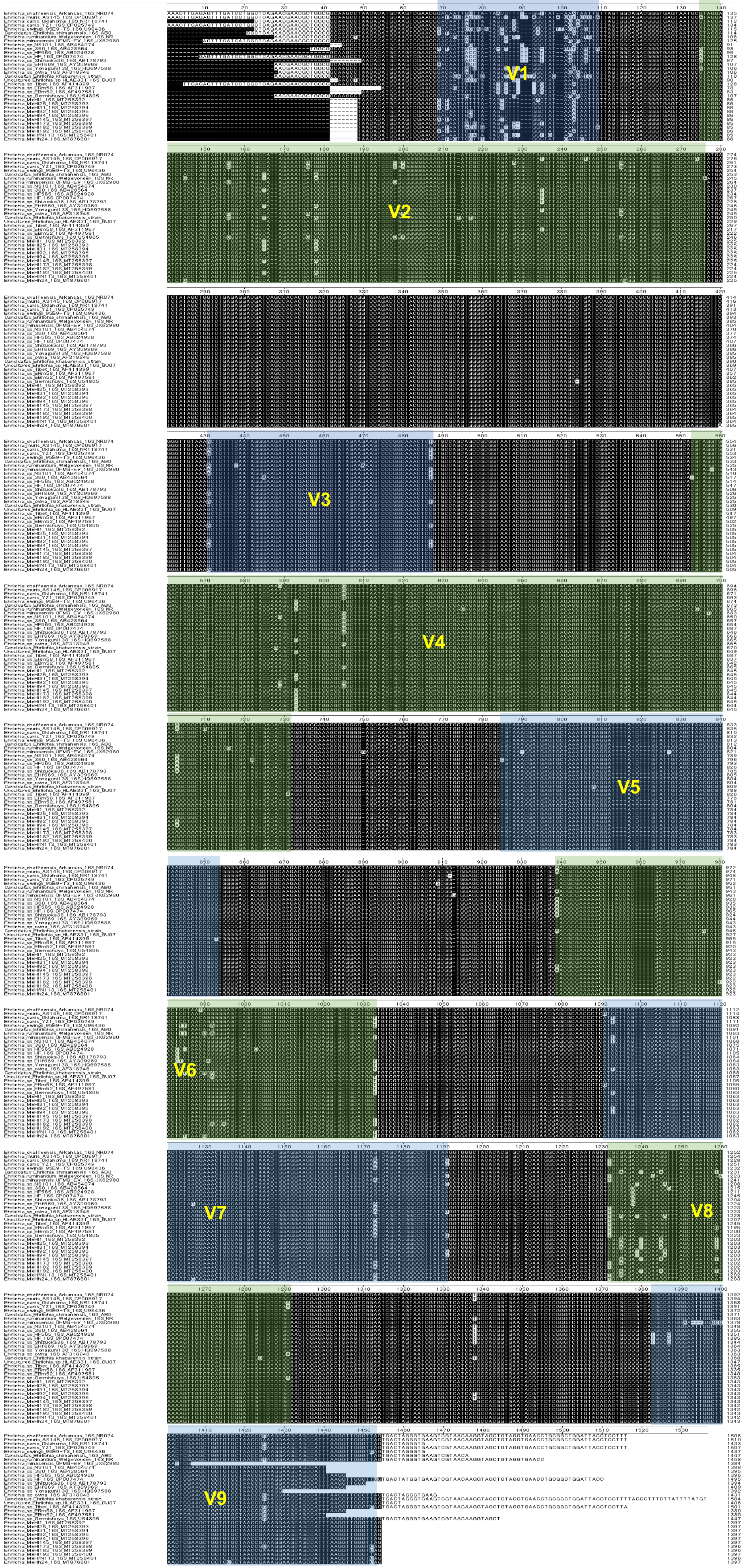

Supplementary Figure S2: Calibration curve of *gltA*-based qPCR assay. The curve was generated using a dilution gradient from 3 x 10^0 to 3 x 10^7 copies/µL in triplicate. The x-axis plots Ct values and the y-axis plots log of standard DNA copy number. The limit of detection was estimated to be three copies.

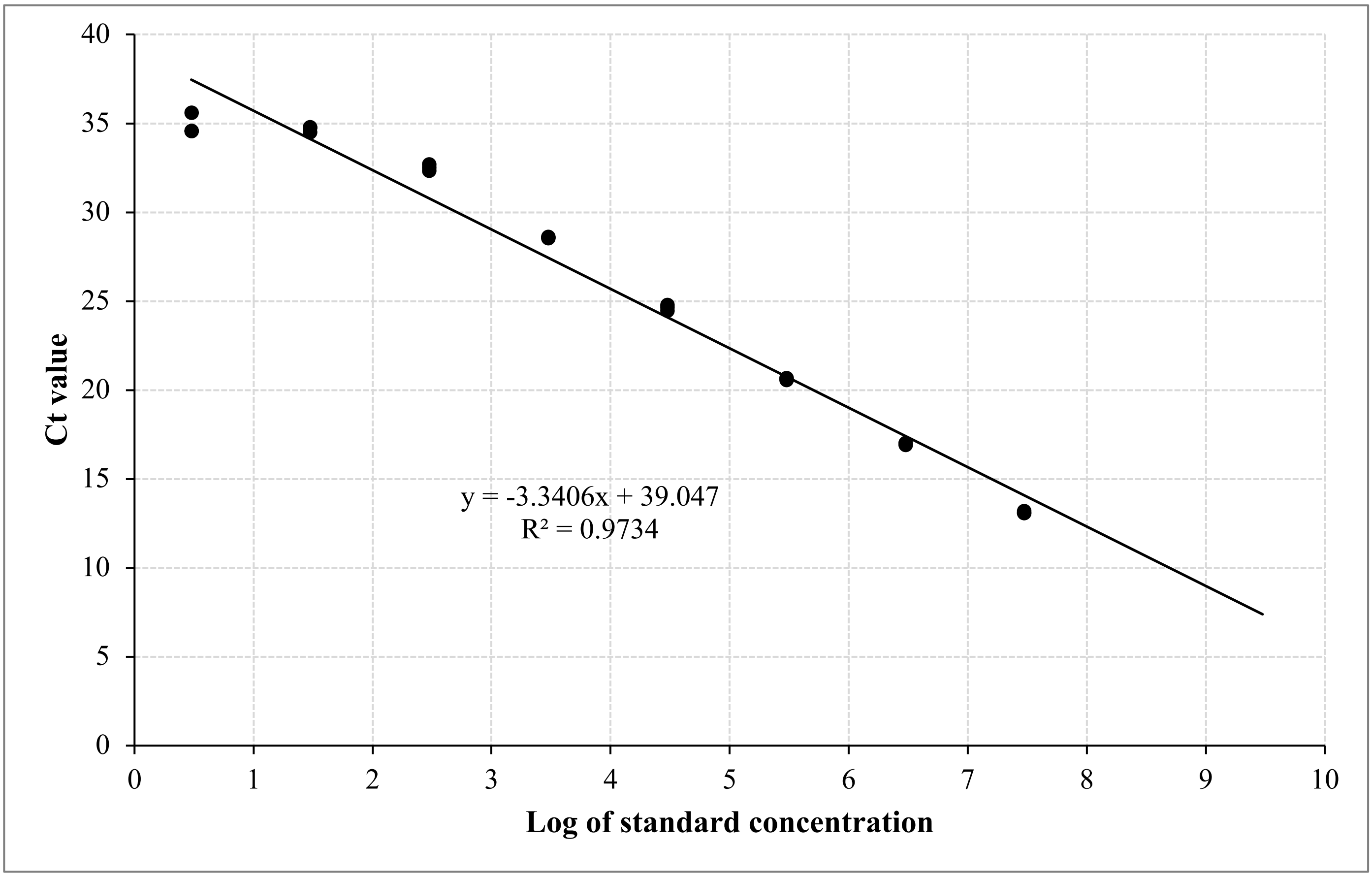

Supplement: Supplementary file 1 — Supplementary Information. [file 41598_2020_80690_MOESM1_ESM.pdf]
